# “Best care on home ground” versus “elitist healthcare”: concerns and competing expectations for medical tourism development in Barbados

**DOI:** 10.1186/s12939-015-0147-1

**Published:** 2015-02-03

**Authors:** Rory Johnston, Krystyna Adams, Lisa Bishop, Valorie A Crooks, Jeremy Snyder

**Affiliations:** Department of Geography, Simon Fraser University, Burnaby, Canada; Faculty of Health Sciences, Simon Fraser University, Burnaby, Canada; Faculty of Medical Sciences, University of the West Indies, Cave Hill, Barbados

**Keywords:** Medical tourism, Health equity, Barbados, Focus groups, Thematic analysis, Health services, International medical travel, Caribbean, Qualitative methods

## Abstract

**Introduction:**

Many countries have demonstrated interest in expanding their medical tourism sectors because of its potential economic and health system benefits. However, medical tourism poses challenges to the equitable distribution of health resources between international and local patients and private and public medical facilities. Currently, very little is known about how medical tourism is perceived among front line workers and users of health systems in medical tourism ‘destinations’. Barbados is one such country currently seeking to expand its medical tourism sector. Barbadian nurses and health care users were consulted about the challenges and benefits posed by ongoing medical tourism development there.

**Methods:**

Focus groups were held with two stakeholder groups in May, 2013. Nine (n = 9) citizens who use the public health system participated in the first focus group and seven (n = 7) nurses participated in the second. Each focus group ran for 1.5 hours and was digitally recorded. Following transcription, thematic analysis of the digitally coded focus group data was conducted to identify cross-cutting themes and issues.

**Results:**

Three core concerns regarding medical tourism’s health equity impacts were raised; its potential to 1) incentivize migration of health workers from public to private facilities, 2) burden Barbados’ lone tertiary health care centre, and 3) produce different tiers of quality of care within the same health system. These concerns were informed and tempered by the existing a) health system structure that incorporates both universal public healthcare and a significant private medical sector, b) international mobility among patients and health workers, and c) Barbados’ large recreational tourism sector, which served as the main reference in discussions about medical tourism’s impacts. Incorporating these concerns and contextual influences, participants’ shared their expectations of how medical tourism should locally develop and operate.

**Conclusions:**

By engaging with local health workers and users, we begin to unpack how potential health equity impacts of medical tourism in an emerging destination are understood by local stakeholders who are not directing sector development. This further outlines how these groups employ knowledge from their home context to ground and reconcile their hopes and concerns for the impacts posed by medical tourism.

## Introduction

The term ‘medical tourism’ is used to describe the practice of individuals traveling internationally with the intention to access medical care. This practice is reported to be growing in popularity, indicated by the increasing interest among medical facilities and healthcare providers to market and cater to foreign patients looking to access more affordable, locally unavailable, or more timely access to medical care [1,2]. In contrast to patients referred out-of-country by their domestic health system, medical tourists direct their own course of care and purchase it out-of-pocket.

The increasing visibility and popularization of medical tourism has raised numerous concerns regarding the impacts of the practice on equitable health service delivery and system development. The influx of additional, private-paying international patients is thought to reduce access to and quality of care for local patients by increasing the demand (and thereby costs) for treatments and by further incentivizing migration of health workers from the public sector to better paying private facilities that are primarily located in urban centres [3,4]. At a systemic level, the capital and labour used to develop new private secondary and tertiary care facilities, often in part publicly subsidized, are arguably inefficient uses of scarce health resources that could be more effectively used in primary care settings more in line with the needs of local populations [4].

In light of these concerns above, this article examines the perspectives and issues raised by local citizens and health workers in Barbados, a country seeking to become a medical tourism destination. We do so in order to better understand the local concerns and expectations surrounding medical tourism among stakeholder groups who are not directly overseeing or influencing the development of the sector and who are commonly identified in existing analyses of medical tourism as those directly impacted by its development. Their perspectives serve to inform and complicate the largely speculative, health system-level conceptualizations of the impacts of medical tourism on health equity summarized above (for additional discussion, see [3,4]) by grounding these debates within the localized context of the experiences and expectations of existing users and workers of the Barbadian health system. This approach allows us to explore the strengths and limitations of existing health equity concerns while identifying additional impacts for further consideration.

This consultation builds upon a previous study examining the early planning for medical tourism in Barbados, as well as our wider work consulting patients, physicians, caregivers, and regulators in the Canadian context about medical tourism (e.g. [5-8]). Together, these stakeholders play critical roles in medical tourism as it is actually practiced and are able to provide important insights into the potential challenges and opportunities it poses to the development and operation of health systems.

### Medical tourism in the Caribbean

Many Caribbean countries have recently demonstrated an interest in pursuing medical tourism as a development strategy [5,9,10]. As tourism dependent states, Caribbean countries are especially vulnerable to fluctuations in the global economy due to their powerful impact on the number of tourists traveling for leisure [11]. While policies directly supporting medical tourism have historically been limited in the Caribbean (with the exception of Cuba), the sector has recently been promoted by many Caribbean governments as an appealing means of expanding tourism-oriented economies in a way that builds on existing tourism infrastructure [9,10]. Demonstrating a regional expansion of interest in exporting health services internationally, many Anglophone Caribbean countries have been involved in the creation of policies, hosting of conferences, and/or development of facilities for medical tourism [9,12].

Projects currently being discussed or pursued in the Anglophone Caribbean include the Health City, Cayman Islands development, a 104 bed hospital staffed by international health workers and focused on the American patient market that began operating in early 2014 [13]. Plans by American doctors to build a new facility in Montego Bay, Jamaica that will primarily treat medical tourists have been well received by the national government, while Turks and Caicos has discussed plans to attract foreign patients by marketing surgical services to international patients at two existing hospitals [14,15]. Additionally, governments in Bahamas, St. Kitts and Nevis, and Grenada are all reportedly exploring their options for developing medical tourism industries [9]. All of these island economies heavily rely on recreational tourism for foreign exchange to fund their public services, including healthcare [11]. As such, medical tourism has been presented as one particularly appealing avenue for economic development given its reputation for generating large revenues and creating high quality employment [9,16]. This dominant discourse, informed by typically inflated projections of the industry’s growth potential and a narrow liberal-economic rationale, regularly neglects to incorporate concerns regarding potential negative impacts of medical tourism on healthcare systems and health equity more generally [17,18]. This may be particularly true in the Caribbean region where research has demonstrated the prioritization of tourism policies and limited involvement of local stakeholders in the development of existing tourism infrastructure [11,19].

Barbados, the most easterly island in the Caribbean with a population of approximately 280,000 [20], is an Anglophone Caribbean country actively working to expand its medical tourism sector. Tourism is among the largest economic sectors, with tourism contributing 11% of the country’s total gross domestic product and directly employing approximately 14,500 Barbadians in 2013 [21]. In contrast, the medical tourism sector in Barbados is currently very small. One facility, the Barbados Fertility Centre, has had great success with recruiting the majority of its patients internationally, primarily from the United Kingdom, Canada, the United States, and other Caribbean countries [10]. The clinic has served as a proof of concept for medical tourism’s fit with the country and has contributed to the Barbadian government’s plans to develop additional medical tourism facilities [9]. The largest and most developed of these plans has been the government’s lease of public land home to a long-defunct private hospital (St. Joseph’s Hospital) to the American World Clinics (AWC) company in 2011. AWC plans to build a 105-bed hospital that will be staffed by locally recruited nurses and a rotating roster of visiting American physicians. The facility will be available to local patients with the means to privately pay for their services, but will mostly focus on serving the international market [22]. This novel approach to healthcare delivery is only the latest of many ‘offshore’ services found in the Caribbean, including the medical education, banking, and gambling sectors [23,24]. While construction has yet to begin, the project is reportedly still proceeding and remains the biggest medical tourism proposal to be actively pursued in Barbados and among the largest in the Caribbean to date [22,25,26].

Here we draw on discussions with local citizens and healthcare providers in Barbados in order to better understand the context in which medical tourism is being pursued in the country and local perspectives on its potential impacts, with a focus on their implications for health equity. Barbadian citizens currently have universal access to healthcare, provided through publicly funded and managed facilities that include primary care polyclinics and the Queen Elizabeth Hospital [20]. This latter facility is the only hospital capable of providing comprehensive tertiary care on the island [27]. While the universal public system provides a common safety net for all Barbadian citizens, many access care in the private sector [20]. Private primary care is greatly preferred by citizens with financial means necessary to pay for it, with 50% of all primary care provided delivered through the private healthcare system [28]. Additionally, there is a small private hospital used by local medical consultants for privately-purchased, non-intensive services [20]. Despite a strong presence of private medical care for primary care and elective treatments, there are no private providers that offer comprehensive tertiary care, with all emergencies necessitating referral utilizing the public hospital [20].

While the Barbados Fertility Clinic is the only facility in Barbados currently marketing to international patients outside of the Caribbean, regional patients (typically from smaller, proximate islands) regularly purchase health services at private and public medical facilities in Barbados [5]. Barbadians also travel throughout the region to access healthcare, both through public cross-border care arrangements and medical trips that are privately financed through insurance or out-of-pocket payments. However, the majority of citizens accessing healthcare off the island do so at extra-regional facilities, often in the United Kingdom or Miami [27]. Due to the relatively small size of Barbados and resulting limitations to the provision of specialized care, international patient mobility has become a norm in this context. There is also a parallel outflow of health workers, with high levels of nurses in particular emigrating from the country to earn higher incomes elsewhere, resulting in nursing shortages and corresponding challenges in providing effective and efficient health services [29].

As a tourism-dependent small island state, Barbados provides an example of a country with a vulnerable, service-dependent economy working to diversify its tourism market. As such, the findings of this analysis are likely to be relevant to other countries in similar economic circumstances that are looking to grow their medical tourism sectors, especially other Anglophone Caribbean nations. By seeking Barbadian citizens’ first-hand perspectives on the development of a local medical tourism industry, this paper offers grounded insights into the complicated economic and health equity considerations posed by the growth of medical tourism.

## Methods

The core research question informing this study is: how do Barbadian citizens and health workers understand medical tourism and what concerns and expectations do they have for the sector? Focus groups were chosen as the qualitative data collection method best suited to explore this question as they encourage the inclusion of a wide range of nuanced perspectives on a topic, especially among participants who may not have expert knowledge with the topic at hand but can bring broadly relevant knowledge to bear on the question [30,31].

### Participant recruitment

Two focus groups were planned for May, 2013. Each focus group was structured to involve a distinct group of stakeholders; the first comprised Barbadian citizens with no professional ties to the health services sector and the other made up of Barbadian nurses. We originally intended for the health worker focus group to include a mix of health workers in different professional roles, but with the exception of one physician who expressed interest, only nurses responded to our calls for participants. We then decided to conduct the focus group with only nurse participants to concentrate the focus of the group on issues raised and perspective offered by a single, front-line care profession. Participants were recruited through a mixture of channels, including newspaper advertisements, community email lists, posters in public spaces, and snowball recruitment among participants. All recruitment materials stated the researchers’ institutional affiliations, the goal of the study, and provided contact information for the investigator who directed and managed on-site recruitment. The study protocol was reviewed and approved by the research ethics boards of Simon Fraser University and the University of the West Indies (Cave Hill) prior to participant recruitment.

Upon expressing interest in the study, potential participants were provided a document that further outlined the study goals, logistics of data collection and analysis, the risks and benefits of participation, and a copy of the consent form to review prior to the focus group. Eligible participants for the citizens focus group were required to 1) be over the age of 18, 2) be a Barbadian citizen, 3) have utilized the public health system in the past five years or have a household family member who has done so, and 4) not be involved in the provision of health services in the country. The nursing focus group used the same eligibility criteria as points 1 and 2 above, but also required participants to have been employed as a nurse in the public health system within the last five years.

### Data collection

The focus groups each ran in the early evening for 1.5 hours and were held in a hotel meeting room. In recognition of the evening schedule, participants were provided with a meal. Nine participants, five women and four men, attended the citizen focus group and seven participants, five women and two men, attended the nursing focus group, meeting the prospective target of 6–10 participants per group. All participants in both focus groups had completed post-secondary education and, with the exception of one member of the ‘citizen’ focus group, were also all employed in professional positions. The mean age of the citizen focus group was 50 years (median of 51 years), with the mean age of nursing participants being 48 years (median of 48 years). No participants withdrew from the focus groups once enrolled.

Both focus groups were structured using the same format with four core probes (general and overarching questions) punctuating what was otherwise an open conversation. Additional sub-questions were only used in cases where participants required additional prompting or re-framing to begin discussing the current probe. Participants were given a brief introduction by the co-moderators at the beginning of the evening that served to introduce the investigators, the research topic, and outline ground rules for focus group interactions. Moderators provided additional questions when a line of conversation concluded or in cases where clarification was required and only intervened in the conversation in cases where a participant indicated a desire to speak but was unable to break into an exchange. Additionally, at the end of a lengthy exchange covering many topics, the lead moderator would provide a verbal summary of what they understood the consensus to be as well as any unresolved disagreements that remained in order to invite clarification and correction from the group. Focus group (co-)moderation was shared between the first two authors, with each taking the lead role for one focus group. All of the participants were provided with a small gift valued at USD $10 at the conclusion of the focus group to thank them for their time and contribution to the study.

### Analysis

Both focus groups were digitally recorded and transcribed verbatim, during which participants’ identities were anonymized. Following transcription, both transcripts were separately reviewed by the first three authors who each created their own interpretation of a comprehensive and robust coding scheme. Subsequently, a meeting among the investigators integrated these schemes by comparing suggestions and resolving redundant and outlier coding categories in order to generate a single scheme. The transcripts were then uploaded to NVivo [32], a qualitative data management program, and coded by the first and second authors using this scheme. After the transcripts were independently coded, inter-rater reliability was assessed in order to highlight any outlier interpretations between the coders by reviewing the coding agreement report generated by NVivo. There was a median value of 87% agreement (mean = 86%) across 26 discrete codes, ranging from 62% for the two lowest codes to 98% for the two highest. The first and second authors thoroughly discussed their difference in interpretation by reviewing and recoding each piece of text within codes with low-agreement in order to harmonize interpretation. As a final step, the coded text was reviewed by the first three authors in order to identify cross-cutting themes and issues present across both focus groups and understand their relationships with one another. These themes along with their associated coding extracts were shared with the full team of investigators in order to confirm interpretation of the findings. In the section that follows we present the findings of the thematic analysis, integrating findings from both the citizens and nurses.

## Results

Overall, both focus groups touched on many of the same issues and shared a similar tone and perspective on the opportunities and challenges posed by medical tourism. The nurses spoke in both their capacity as Barbadian citizens and users of the health system, bringing their experiences as health care professionals to some, but not all, issues. As such, there was a great deal of thematic convergence across the focus groups, both being broadly supportive of the idea of a larger medical tourism sector in Barbados. Both groups approached medical tourism as an economic benefit, a means to increase local access to medical specialties not available on the island. However, the conversations also raised numerous concerns about how the sector could negatively impact the country and its health system. The nursing focus group additionally identified potential positive and negative outcomes stemming from medical tourism in relation to employment of nurses, Barbados’ medical culture, and nursing training. Thematic analysis identified four cross-cutting focus group themes that clarify the sources, bounds, and intersections of the expectations and concerns that were raised. Here we organize these themes into the two broad domains of ‘Local Setting’ and ‘Competing Expectations’. Themes pertaining to ‘Local Setting’ were largely descriptive and worked to situate medical tourism in a localized understanding of the Barbadian context. In contrast to this are the themes associated with ‘Competing Expectations’ that highlighted tensions in ideas around how the developing medical tourism industry and its impacts might unfold in Barbados. These domains and their themes are explored in detail below. Unless specifically noted, the themes and issues raised were common to both focus groups and where quotes are provided, the focus group source is noted in brackets.

### Local setting

The first of the two major thematic domains that arose across the focus groups were ideas and issues pertaining to Barbados as a dynamic tourism-dependent setting that a larger medical tourism sector would be developing from and interacting with. As a setting, Barbados’ long experience with and reliance upon tourism was critical in participants’ understandings of the potential economic and social impacts medical tourism creates for the country. Likewise, the longstanding international networks that connect Barbados’ patients and health workers to hospitals abroad in terms of training, employment, and care seeking, served to normalize medical tourism for the participants and offer a lens with which to interpret their understanding of and expectations for the sector.

### Health services export as niche form of tourism

The significant role of recreational tourism in Barbados’ economy and society served as the most common point of reference for participants across both focus groups and situated their understanding and expectations of medical tourism. Participants noted that medical tourism was one among a host of diversification strategies for the country’s tourism sector being discussed in public forums alongside sports, heritage, and eco-tourism. Both focus groups noted that public conversation about medical tourism has been ongoing but sporadic, with government press releases and investor plans for facilities triggering ‘buzz’ in the media for developments that had yet to be realized. Altogether, medical tourism was not a pressing concern for participants and was situated unremarkably as just one form of tourism within the ongoing public conversation about tourism diversification and development. Because the existing tourism industry served to prime participants’ initial understandings of medical tourism and their expectations for its development, medical tourism was initially framed as an economic development issue and only secondarily understood as one concerning healthcare.

Participants of both focus groups perceived that Barbados has an international reputation for safety and privacy and that this reputation is a critical support for its tourism sector. However, medical tourism was seen as a disruption to maintaining the country’s reputation for safety due to the regulatory and monitoring challenges the sector poses. One participant summarized this concern:

*…if you had a serious issue like you know some virus broke out because of whatever malpractice issue … then all of a sudden no one is going to come here anymore for any medical tourism and then they may also impact on the general tourism, the sun, sea and sand because like say in Barbados you allow person to come there for treatment and they die.* (Citizens)

Mirroring participants’ emphasis on interpreting medical tourism through their understanding of the recreational tourism sector, the potential negative impacts of health services export included wider economic impacts, not just those of healthcare and health equity.

### Existing international healthcare connections

The stories shared by participants demonstrated that there is a popular awareness that Barbados is deeply integrated within existing international networks of care, in terms of Barbadians traveling for treatment, Barbadian hospitals providing care for patients in the surrounding region, and the well-established international routes of health workers moving to and from the country. Existing outbound medical travel by Barbadians was raised in both focus groups and was more relevant to participants than inbound medical tourism, summarized here by one participant stating

*I really don’t think that medical tourism is much on people’s radar here as something that is very prevalent or touches people’s lives from day to day you know, I don’t think most people even know to what extent that it happens …I think the average person is more, has more on their radar leaving for a medical procedure if it’s necessary than other people coming.* (Citizens)

Discussions of mobility among Caribbean patients highlighted existing regional outbound healthcare networks. One participant noted that *“I can envision people, wealthy Barbadians who would go to Miami, who would go to New York and those kind of places… I can see some of them remaining here, if there is such a facility and that saves us foreign exchange (Nurses).”* The idea of patients traveling abroad for medical care was, thus, a familiar one.

Further discussion among participants indicated that a factor influencing utilization of non-local health services was a desire to see specialists with higher volumes of patients, and thereby expertise, than is possible locally. For example:

*I think a lot of the issue with having the best care is not necessarily somebody here being unable to give you an opinion or unable to do the procedure, but when you look for example at Miami Children’s and you think about the fact that if you have to have something done for you* [sic] *child here who has a relatively rare something that the doctor here may have seen twice, whereby you’ve got 500 children going through Miami every year […] the level of expertise is always going to be different.* (Citizens)

Although there was recognition that the development of a medical tourism sector could enable Barbadians to access care locally that is not currently available, there was concern among some participants that they would not want the presence of such services to lessen their access to procedures abroad that are funded by government or private insurance. *“I would be very upset to be forced [by my insurance company] to go to [a renovated] St. Joseph Hospital [in Barbados built for medical tourists] because it’s now available here but it’s not necessarily the best care”* (Citizens)*.* Participants were keen to ensure that the development of a domestic medical tourism sector would not erode their own access to health services both at home and abroad.

While discussions on existing patient mobility mainly focused on Barbadians traveling to international destinations, inbound regional care networks were also mentioned as playing an important role in providing healthcare to Caribbean patients. Discussions on regional patient mobility particularly emphasized Barbados as an existing destination for patients from smaller nearby islands such as Antigua. Some participants indicated that the development of medical tourism in Barbados should consist of a concerted effort to increase the regional patient flow to Barbados. This was seen as a measure to enhance access to the *“best care on home ground”* (Citizens) for Caribbean patients by using the income generated from the increased provision of healthcare regionally to support health worker specialization and technological innovation locally; *“If technologically it is more advanced and more effective and that is what you need for your health, if it is accessible, then I think we should make a way to make it available [to other Caribbean citizens]”* (Citizens). Participants thereby expanded their understandings of scope of medical tourism beyond the existing local narrative of inbound Americans, Europeans, and Canadians to include a regional focus.

### Clarifying expectations

The focus groups explored different dimensions of an expanded Barbadian medical tourism sector, which were informed by participants’ knowledge of and exposure to medical tourism in Barbados thus far, specifically the Barbados Fertility Clinic and the planning for the St. Joseph Hospital renovation by American World Clinics. Discussion of these two very different projects, in terms of scale, ownership, range of specialization, and system integration clarified participants’ expectations for what shape medical tourism in Barbados might take, the potential for local economic benefits, and the facilities’ degree of integration with the existing healthcare system. Discussions also highlighted some tensions between their expectations around the potential benefits to and negative impacts on the Barbadian healthcare system. For example, participants debated the system changes emerging as a result of interactions between medical tourism and the existing health system in relation to local regulations, professional associations, healthcare professionals, and local patients. In this section, we examine two distinct groupings of expectations, those pertaining to the scope and structure of the sector and those pertaining to its impacts on the health system.

### Scope and structure of a medical tourism sector

The ongoing planning to develop foreign-owned hospitals in Barbados that will primarily staff non-local specialists and export their services to international patients (i.e. offshore medical services) was generally accepted by participants, but they did raise concerns about the degree of meaningful integration with the local economy. Some participants drew unfavourable parallels with all-inclusive recreational resorts in the Caribbean. Participants critiqued this tourism model for generating (mostly low-skill) employment for locals but few additional economic benefits for the host communities. There was shared agreement that medical tourism facilities had no obligation to reinvest profits locally but should in turn not be granted public subsidies such as tax concessions. It was also generally agreed among participants that any new medical facilities in Barbados should be affordable to local users such that they might directly benefit from their presence. Overall, a skeptical current ran through both focus groups as to the scale of benefits for Barbados in hosting foreign-owned hospitals:

*I don’t think that Barbados will benefit to the extent that we may think, that you may have a lot of spin offs, you can have thousands of jobs being generated and that sort of thing. I don’t want to sound too pessimistic, but I like to err on the side of caution… the bulk of money will be staying over there [with international investors], it won’t be here. Yes we will get something, but it will be the crumbs.* (Nurses)

Participants wondered if and how any new medical tourism facilities might rely on, or operate in complete distinction from Barbados’ public healthcare facilities. Participants across both focus groups almost unanimously agreed that any new facilities should be self-sufficient in delivering the full spectrum of care they require, including critical care for emergent complications, and that they should not require any support or services from public hospitals and clinics. Because Barbados currently has only one (public) hospital capable of delivering tertiary care, participants did not want any private facilities introducing additional burden to the public system, one stating:

*[W]e don’t want a situation where the new facilities impact on the QEH. They should be able to contain themselves. I think they should have an ICU [intensive care unit] and if there are any complications they should be able to maintain or at least…so that it doesn’t impact on general healthcare.* (Citizens)

In the citizen focus group, some participants considered the potential for private facilities arranging to pay for any public care services they might need in the event of an emergency, but this was rebutted by other participants who thought any private imposition on the already burdened public system was unacceptable as it would directly translate into reduced access for local patients.

Finally, participants in both focus groups articulated a vision for what they thought would be the most successful and well received form of medical tourism among Barbadians. At first consideration, participants closely associated medical tourism with cosmetic surgeries, for which there was widespread acceptance for a well-developed export sector. More generally, participants encouraged a form of medical tourism to Barbados that focused on elective, low risk specialties and developing an internationally renowned niche.

*[S]o what I’m say is instead of doing all these things if you were to build a facility and you were to advertise to the world, right that we have this facility we do knee replacements, we do hip replacement or we do something or the other and pick out half a dozen thing that you would specialize in so if you want a brain tumour work on, go somewhere else we don’t want you come here because we’re not involved in that.* (Citizen)

Relatedly, some participants expressed a preference for local physicians forming the core of a Barbadian medical tourism industry through cultivating specialist niches among local providers instead of hosting large, foreign owned and staffed medical facilities. “*What are we willing to invest in our people and getting our country up? Why not look at it that way… Why bring in somebody to run it for us?*” (Nurses)

### System disruption versus system improvement

Participants raised concerns that medical tourism could work to loosen regulatory standards for healthcare providers and weaken the nurses’ labour union. Discussion about professional oversight indicated that some participants were concerned that the potential for reliance on foreign healthcare professionals to provide the labour in new medical tourism facilities could overwhelm or elude professional regulation, one saying *“are [foreign healthcare providers] just going to walk all over or circumvent the Barbados Medical Council or Nursing Council?”* (Nurses)*.* Participants in the nursing focus group emphasized their expectations that local training and licensing requirements should apply to foreign trained care workers and questioned the potential for pressures by international investors in private medical tourism facilities to change the standards set by these professional bodies. One participant raised the strong unionised labour tradition in Barbados, including nursing staff, and a concern that nurses could be impacted *“if the owners of these facilities do not want persons who are unionised”* (Nurses). This concern was framed by discussions of past instances of resistance to organized labour among international corporations set up in Barbados. Taken together, the focus groups demonstrated that there is concern about how offshore medical facilities would integrate with existing local professional institutions and labour norms.

Participants expressed an expectation that the development of a medical tourism industry would improve the existing healthcare system. It was hoped that medical tourism would provide local patients access to a wider range of specialties and services while also offering opportunities for knowledge-exchange between international and local care providers. This hope was partly informed by existing experiences where there have been cases of “*a patient who has a special need and they bring down their specialised doctor, when the procedure is being done, both parties are present; the [doctor] from Barbados and the specialised doctor. So there is a little training goin’ on”* (Nurses). However, participants were also concerned that foreign health workers at new medical tourism facilities may serve to alter local patients’ health and healthcare expectations. This concern is summarized by the following quote:

*It’s going to be a little bit of an issue for our medical professionals here. Because if you are going to get a real specialised person that is going to say ‘the doctor in the West Indies says that after 5 minutes you are dead, that is a lie, I can resuscitate you in 2 minutes or in half a second.’ So then the average person is going to say ‘oh well these doctors and medical people here [in Barbados] don’t really know what they are doing, I want outside treatment.* (Nurses)

The expectation for collaborative, in-person engagement among health professionals that is suggested by the particular form of medical tourism participants advocated for in Barbados was thus seen to be in tension with the potential for conflict between clinical cultures and competition for patients.

Participant discussions highlighted concerns about the potential loss of healthcare workers in the public sector due to the emergence of medical tourism facilities as they expected the medical tourism sector would be a more attractive employer for health workers than the public sector. One participant expressed this concern in saying:

*if the whole industry tends to grow, [and] if the facilities are set up in such a way that locals can’t really use the services and the doctors are attracted to go and work with these facilities and then locals don’t have access… to qualified doctors. Because obviously the most experienced ones are going to be attracted to go and work privately and they may not spend a lot of time working for the QEH.* (Citizens)

Alternatively, participants also framed the medical tourism industry as a potential catalyst for improved working conditions in the public sector, expecting that increased competition from the private sector could *“force our government, force our nursing governing body to review, to respect us, to encourage to continue education and give the opportunity for us to further our education without any further harassment or obstacle”* (Nurses)*.* The potential for new private facilities to increase local demand for nurses and introduce direct exposure to different clinical management styles raised hope among the nurse participants for improved working conditions, pay, and overall respect for the nursing profession in Barbados. However, this was tempered by concerns that foreign owned facilities might plan on importing nurses to provide specialties rather than employing locals and providing employment and training opportunities.

## Discussion

Much of the academic work examining medical tourism to date has focused on tracing the *potential* health equity implications of patients electively tapping into international networks of medical care beyond their home health systems. This early body of literature is largely focused on the potential impacts posed to abstracted health systems, including both systems that are ‘sending/losing’ and those ‘receiving/gaining’ patients (e.g. [12,16,33]). Only recently has scholarly research started to produce empirical accounts of the experiences of medical tourists, medical tourism facilitators, healthcare workers, and health system administrators in both ‘home’ and ‘destination’ countries (e.g. [7,34-36]). Here we have used an empirical account to begin addressing another key existing gap in the medical tourism literature, namely how medical tourism is perceived by citizens and health system users in countries at a nascent stage of sector development. In the sub-sections that follow we examine the significance of the findings shared above and the implications they hold for further empirical investigation about medical tourism.

### Dynamic and networked destinations

The focus group findings illustrate how Barbados’ medical tourism initiatives are being developed in a place that is enmeshed in existing international networks and relationships of trade, care, and migration. Participants highlighted a number of existing considerations that inform their understanding of medical tourism and their hopes and concerns surrounding the sector that reflect this dynamism. Firstly, the recreational tourism sector, with its large and existing role in the economy, emerged as an important part of national identity in the focus groups and served as a regular point of reference for framing what kind of national economic development is viable and welcome. The enormous economic value of tourism recognized by both focus groups as critical to sustaining the country’s high standard of living, served as a dominant frame that had participants consider medical tourism as a niche form of tourism first and health services export second. This ordering seemingly prompted participants to accept the premise of the industry from the outset and begin working backwards to find the boundaries of where unacceptable interaction and infringement on existing health services begins instead of proceeding from the opposite direction and assuming the practice was unwelcome and working forward to find under what conditions it might be acceptable.

Similarly, other contextual factors familiar to Barbados and the wider Anglophone Caribbean resulted in participants’ ideas about and perceptions of migratory healthcare as rather unremarkable. The extraordinarily high rates of nursing emigration from the Caribbean and the existing regional care networks, both publicly and privately financed, that participants were all familiar with (and some had personally relied upon) contributed to broad agreement across both focus groups that an expanded medical tourism sector is an appropriate fit for Barbados’ future economic development. A key complementary factor underpinning the participants’ ease in conceptualizing the industry on the island was a resigned acceptance of the critical role international trade in services plays in the current Barbadian economy with regard to financial services and recreational tourism. The broad acceptability of exporting health services shared among the participants, however, was quickly contested once they began to explore the potential outcomes in detail. Instead, participants favoured a more tailored vision of medical tourism consistent with Connell’s critique of the Caribbean medical tourism sector [9], as a sector that focuses on the development of niche areas of medical expertise. This finding suggests that new medical tourism developments being proposed in Barbados may be assisted by medical tourism’s indistinct and broad conceptualization among local residents, serving to prevent local critique and pushback until specific (potentially undesirable) projects are well underway.

Finally, participants’ general enthusiasm for incorporating regional Caribbean patients in developing Barbados’ medical tourism sector builds upon Ormond’s [36] call to consider regional ‘complementarities’ in care provision in discussions of internationalizing healthcare Meanwhile, there is little discussion about the potential to grow regional healthcare networks through medical tourism in the policy and public discussion about sector development (see, for example, [22,37-39]). As a development strategy, Barbados would likely benefit from working with its neighbours to better document and formalize existing patient flows within the Caribbean in order to identify what specialties are locally oversupplied and in high demand regionally. While such an approach would not exclude simultaneous efforts to attract patients from outside the Caribbean region, reframing medical tourism so that it consciously includes all international patients and not exclusively those hailing from the ‘Global North’ would encourage the development of health infrastructure that is most relevant to local users. Such a narrative, however, runs counter to the ways in which medical tourism is regularly promoted in the Caribbean and elsewhere by industry groups such as the *Medical Tourism Association*, where the focus is on recruiting patients from the United States and other high-income nations.

### Grounding health equity concerns

Both focus groups raised three of the potential negative health equity impacts of medical tourism that are consistently discussed in the literature: 1) internal health worker migration from public to private facilities, 2) public resources being provided to private facilities to incentivize development, and 3) the emergence of two tiers of quality in medical care [4,40]. Each of these concerns was perceived to be unwelcome, but each to a differing degree according to potential tradeoffs with health system benefits. Small-scale internal health worker migration, particularly in regard to nurses, was not desirable but was largely discussed by participants to be an understandable cost of trading in health services. Participants demonstrated tolerance for private sector competition for nursing labour, with many of the nurses, perhaps unsurprisingly, interested in a wider range of employment and training opportunities locally, higher pay, and a more rewarding work environment than what is currently found in the Barbadian health system. More significantly, some participants’ opposition to local specialists diminishing their participation in the public sector alongside their acceptance of additional private sector competition for nursing labour distinguished between what kind of labour competition emerging from medical tourism is understood to be unacceptable and that which is perceived as fair. Conversely, there was broad agreement among both focus groups that participants did not want any public resources directly supporting the medical tourism industry, especially for foreign-owned facilities, with high resistance to tax concessions for workers’ income or facility profits. This differential weighting of various health equity concerns expressed by participants demonstrates the benefit of exploring the relevance of each concern for particular settings in future research and questions the suitability of framing all forms and instances of medical tourism as inherently prone to inequitable outcomes.

The (un)acceptability of medical tourism contributing to two different tiers of care within Barbados was much less clearly delineated within the focus groups than other health equity impacts that were discussed and caused the fiercest debate among participants. The existing Barbadian healthcare landscape, with a comprehensive and universally accessible public health system co-existing with a small private hospital and many private primary care clinics, informed the conversation around this concern. Because the existing universal public system attenuates any extreme situations of Barbadians being completely denied access to medically necessary care that medical tourists would be able to obtain privately, most participants saw expansion of medical tourism as an acceptable enlargement of the country’s existing private healthcare sector. However, some participants were brought to emotional exchanges at the prospect of hospitals supported by medical tourism exacerbating existing private/public healthcare inequities and inequities between citizens and foreign visitors on the island. Whereas the existing private hospital in Barbados is staffed by local specialists who also serve patients in the public hospital, the potential for a medical tourism facility to offer superior-quality care for local patients because of better amenities and/or more highly trained (foreign) specialists would produce an unacceptable alternate tier of care within the local healthcare system. One participant articulated this mode of medical tourism as “*elitist healthcare*” (Nurses) that would undermine health equity in the country. These concerns, when taken together, articulate a coherent vision of inequity where commercial interests in healthcare override access to equivalent quality care for local patients. This distinction of universal access to *equivalent* quality and range of care versus strictly *equal* financial and temporal access to care is critical in understanding what particular (and inevitable) trade-offs are considered fair in Barbados and what ‘two-tier’ care means across different international contexts.

Finally, the concern raised by some participants that a local, comprehensive-service private hospital made possible by medical tourism might ultimately limit their care options due to private health insurers refusing to continue to reimburse out of country alternatives has, to our knowledge, not been raised in the existing discussions of the health equity impacts of medical tourism. This finding highlights the unknown range of potential disruption to established healthcare norms and patient expectations that new hospitals supported by medical tourism pose. Along these lines, it underscores the need to undertake empirical research in order to capture on-the-ground insights about the complex ways in which medical tourism can positively or negatively impact health equity in specific places.

### Scale and form of internationalizing healthcare

Underlying all of the participants’ views about medical tourism in Barbados was an ongoing negotiation of the form and scale of medical tourism that was seen as an acceptable, if not desirable, fit for the country. Participants were largely pleased with the existing medical tourism activity, namely the Barbados Fertility Centre’s success in attracting international patients, due to the clear economic benefits of increased tourist numbers and the attendant local availability of fertility services. This broad agreement across both focus groups that Barbados should develop a medical service export strategy that relies upon a reputation for excellence in low-risk elective treatments provided by local practitioners was informed both by two of the participants’ core concerns not informed by health equity, namely protecting Barbados’ international reputation and maximizing economic gains for the local economy.

Participant concerns that the financial benefits of medical tourism might leave the island under foreign ownership were raised in relation to examples of unintended consequences from previous foreign investments in Barbados. Nursing participants’ expectations for the strong local nursing and support staff unions to be respected and integrated into the plans of foreign owned facilities raise questions of how equitable labour relations might be supported or undermined by the novel offshore model of medical tourism being pursued in the Caribbean, particularly given earlier instances of acrimonious relations between foreign-owned corporations and local organized labour. Similarly the expectations of both focus groups for Barbados’ health professional associations to be consulted and their certification requirements respected by offshore medical facilities highlight points of potential stress in globalizing healthcare arrangements. While the nursing focus group in particular saw great potential for training opportunities in a foreign-owned facility, participants also raised the example of a previous foreign investment in information technology that did not follow through on meaningful training of local workers that was part of its concessions package, indicating some concern in promised benefits being realized. These expectations highlight investment conditions that might be leveraged by national governments considering foreign-owned medical tourism projects in order to ensure they benefit the existing domestic health system and its workers.

Relatedly, participants saw the potential for offshore hospitals to mirror the negative aspects of all-inclusive resorts in the Caribbean. When coupled with concerns around questions of long-term commitment among foreign investors, wariness of local care providers, and the hesitation to provide tax concessions as an incentive for facility establishment, there are potentially serious roadblocks for public support for offshore medical facilities in Barbados as well as the wider Caribbean. However, these same concerns might be outweighed by participants’ hopes for access to a greater range of care options, employment, and training locally, all of which were repeatedly tied to participants’ preferences for a locally integrated and small scale medical tourism industry. These preferences for the scope and scale of the industry by study participants echo and contribute to Connell’s [9] exploration and critique of the current planning for medical tourism going on in the Caribbean, where he identified the region as likely to be unsuitable for large scale, comprehensive health services export.

### Limitations

While this analysis provides some insight into the perceptions of some nurses and health system users in a specific medical tourism destination looking to expand its industry, it does not capture the outlooks of physicians, patients who solely rely on the private health sector, nor patients who are solely reliant on the public health sector. As such, the range of perspectives shared in the focus groups were limited in their scope to only one professional group and, with regard to education and employment, a relatively homogenous section of health system users who rely on a mixture of private and public health services. However, we do not seek to provide a comprehensive account of the health equity concerns and benefits posed by medical tourism, but instead to both broaden the scope of existing health equity impacts that have been theorized and also explore their relevance among some of the groups that have been framed as those most directly impacted.

## Conclusions

Our motivation for seeking to address the existing research gap surrounding local residents’ understandings of medical tourism in countries seeking to expand this sector is informed by two complementary concerns. Firstly, the growth of medical tourism, especially in the case of patients from high-income countries accessing care in low-income settings, has raised many significant health equity concerns regarding the fair use and distribution of domestic healthcare resources as they are incorporated into, or diverted toward, the international market. Arguments for economic development, complementarities between healthcare systems, existing systemic dysfunction, and prospects for improved care quality in destination countries have all been raised to complicate the ugly aesthetics of (relatively) wealthy foreigners unjustly appropriating scarce health-care resources in poorer nations (e.g. [2,36,41]). This health equity debate is ongoing and important, but arguably requires finer resolution and further grounding. This paper demonstrates the ability for empirical examples of medical tourism development to add nuance to this conversation. In the instance of Barbados, a relatively high-income nation possessing a universal healthcare system, the striking popular image of medical tourism characterised by full-service private hospitals amidst endemic poverty and poor local access to healthcare that has been popularized by cases such as India and Thailand does not hold. However, health equity concerns specific to the Barbadian context do factor into citizens’ considerations regarding the development of this sector and suggest directions in which medical tourism might be least disruptively pursued in small-island contexts.

Secondly, the exploration of the health equity impacts posed by medical tourism has yet to engage with a larger existing debate about what health equity actually is. This debate, in its most crude conception, pits universally normative claims about the definition of good health and its value against culturally relativistic doubts about any truly ‘global’ conception of global health equity (e.g. [42,43]). We argue that global health equity is least constructively conceived as a pre-determined normative goal or vision to be prescriptively satisfied and instead most valuably understood as a political process unfolding at international, national, and sub-national scales, one that meaningfully consults and incorporates the wishes and perspectives of stakeholders at each of these levels. This analysis is a small instance of the kind of consultations that can contribute to health system development that encourages genuinely equitable health outcomes understood to be acceptable and fair by its workers and users.

Finally, while our research findings highlight the importance of gaining contextualized considerations of health equity processes, particularly in relation to international healthcare markets, this research also demonstrates potentially shared experiences and concerns amongst tourism-dependent countries. Participant framing of this industry as another tourism diversification strategy indicates the high degree of interaction between the medical tourism and recreational tourism sectors in a national context that is deeply reliant on visitors. Participant discussions emphasized the importance of recreational tourism on the island and their expectation that the sector be protected and prioritized in policy-making and in any considerations of diversifying the country’s service exports. In these discussions, Barbados the country became Barbados the brand. This framing is counter to many scholars’ calls to move away from the term ‘medical tourism’ in favour of the more solemn ‘international medical travel’ (e.g. [44,45]). The strong relationship, in terms of economic and policy development, between the medical and recreational tourism sectors in tourism dependent locales demonstrates the importance of engaging with the language and literature of tourism when considering the development of the health services export sector, its potential impacts on local and global populations, and the creation of regulations and/or norms within the global industry. This engagement could be of use in better understanding and responding to the vulnerabilities characteristic of tourism-dependent contexts that are developing their health services export sector.
